# Isolation, identification, and susceptibility testing of *Staphylococcus caprae* from dairy goats with mastitis to antibiotics and Chinese herbal medicines

**DOI:** 10.3389/fvets.2025.1690281

**Published:** 2025-12-10

**Authors:** Jie Luo, Yiping Wang, Wenya Zheng, Chaolong Xu, Jianhua Chen, Ben Liu, Xiaoyue Wang, Manxin Fang

**Affiliations:** 1 School of Life Sciences and Environmental Resources, Yichun University, Yichun, China; 2 Yichun University Research Center for Traditional Chinese Veterinary Medicine and Animal Embryo Engineering Technology, Yichun, China

**Keywords:** dairy goats, mastitis, *Staphylococcus caprae*, isolation and identification, pathogenicity, susceptibility testing

## Abstract

**Introduction:**

Mastitis outbreaks in dairy goats at a farm in western Jiangxi Province, have recently imposed significant economic losses on farmers. This study aimed to identify the causative pathogen and implement targeted control measures for early disease eradication.

**Methods:**

Pathological autopsy was performed on diseased goats submitted for examination. Pathological mammary gland tissues and milk cistern fluid were collected aseptically for bacterial isolation and purification. The isolated pathogen was characterized through colony morphology observation, Gram staining, biochemical tests, and 16S rDNA gene sequencing. Pathogenicity was assessed using an animal challenge test in mice. *In vitro* antibacterial susceptibility testing was conducted using 30 Antibiotics (e.g., cefradine, cephalexin, cefuroxime), 18 Chinese herbal medicines (CHMs; e.g., *Rhus chinensis* (Galla Chinensis), *Coptis chinensis* (Chinese Goldthread), *Punica granatum* (Granati Pericarpium)), and 15 phytogenic extracts (e.g., Berberine, Gallic acid, Tannic acid).

**Results:**

A Gram-positive bacterium was isolated from the diseased tissues. Biochemical profiling and 16S rDNA sequencing identified the isolate as *Staphylococcus caprae*. The animal challenge test confirmed the isolate’s strong pathogenicity in mice. Susceptibility testing indicated that the isolate was susceptible to Antibiotics such as cefradine, cephalexin, cefuroxime, and ciprofloxacin, while showing the poorest susceptibility to penicillin among the tested antibiotics. Among the CHMs and phytogenic extracts, *Rhus chinensis* (Galla Chinensis), *Coptis chinensis* (Chinese Goldthread), *Punica granatum* (Granati Pericarpium), Berberine, Gallic acid and Tannic acid demonstrated superior antibacterial efficacy.

**Discussion:**

This study provides multiple therapeutic and preventive options (CHMs, Antibiotics, phytogenic extracts) for mastitis control on this farm. It also offers valuable insights into the identification and treatment of mastitis in dairy goats, contributing to safeguarding goat health and ensuring dairy product safety.

## 1 Introduction

The global dairy goat industry is predominantly concentrated in Asian countries, significantly improving livelihoods for low- and middle-income populations in the region. Although countries surrounding the Mediterranean Basin in Europe possess only 5.1% of the world’s dairy goat population, they contribute 15.6% of global goat milk production, playing a critical role in local economic development (1). Compared to bovine milk, caprine milk exhibits superior lipid digestibility, lower allergenicity, and higher mineral bioavailability. It is also rich in conjugated linoleic acid, which plays significant roles in immune function and growth regulation (2). With growing global market demand for goat milk products (3), the health status of dairy goats directly determines the industry’s economic returns. However, the prevalence of subclinical mastitis in dairy goats ranges from 30 to 50%, leading not only to reduced milk yield and deteriorated milk quality but also potentially causing systemic infections. This impacts animal welfare and market value, resulting in substantial economic losses (4). In addition, raw goat milk may be a potential source of the spread of antibiotic-resistant pathogens between animals and humans, threatening the environment and human health (5). Consequently, mastitis has emerged as the core bottleneck constraining the sustainable development of the dairy goat industry.

Subclinical mastitis in dairy goats represents a costly disease within the caprine dairy industry, caused by complex polymicrobial infections involving bacteria such as Coagulase-negative staphylococci (CNS), *Staphylococcus aureus*, *Escherichia coli*, *Streptococci*, and *Mycoplasma*. Notably, the predominant pathogens vary significantly across regions and farming systems (6–8). While tests including somatic cell count (SCC), the California Mastitis Test (CMT), and infrared thermography are considered useful farm-level screening tools, their reliability remains insufficiently validated (9). These methods also suffer from limitations such as time consumption and diagnostic inaccuracies. Precise identification of the dominant pathogens is therefore a prerequisite for effective control. For mastitis management, antibiotics remain the primary preventive and therapeutic approach. However, indiscriminate use contributes to the spread of antimicrobial resistance and drug residue issues. CHMs and phytogenic extracts are derived from nature, have low residues, and demonstrate excellent antibacterial and antifungal potential (10–12). Nevertheless, the lack of systematic antimicrobial susceptibility data for dairy goat mastitis pathogens and the unclear mechanisms of action of these alternatives restrict their clinical application.

*Staphylococcus caprae* (*S. caprae*), a coagulase-negative staphylococcus, was first isolated and systematically described from goat skin and dairy products by Devriese et al. (13). Subsequent studies have demonstrated its ability to induce various human diseases, including dermatitis, arthritis, osteomyelitis, endocarditis (14), neonatal sepsis, and meningitis (15), suggesting its potential as a zoonotic pathogen.

In this study, tissue lesions were examined via hematoxylin and eosin (H&E) staining of pathological samples. Pathogenic bacteria were isolated, purified, and cultured, followed by identification through Gram staining, biochemical tests, and 16S rDNA sequencing analysis to determine the predominant pathogen causing mastitis in dairy goats at a farm in western Jiangxi Province. The pathogenicity of the isolate was assessed using a mouse challenge model. Furthermore, *in vitro* antimicrobial susceptibility testing was conducted to compare the inhibitory effects of antibiotics, CHMs, and phytogenic extracts. This study aims to evaluate the antibacterial performance of drugs, guide precise clinical medication, and lay the foundation for the potential application of natural herbs as alternatives to antibiotics.

## 2 Materials and methods

### 2.1 Pathological samples

Mammary tissue and milk cistern fluid were aseptically collected from a mastitic dairy goat submitted by a farmer from a farm in western Jiangxi Province. The affected goats primarily exhibited udder atrophy, characterized by significantly reduced size, palpable hard masses, and absence of milk secretion. The collected samples were stored at −80 °C.

### 2.2 Hematoxylin and eosin staining

Mammary tissue samples were rinsed with physiological saline, fixed in 10% formalin solution, and embedded in paraffin. Paraffin-embedded tissues were deparaffinized through graded alcohols and rehydrated in water. Subsequently, sections were cut and stained with H&E (Solarbio Science & Technology Co., Ltd., Beijing, China). Stained sections were observed under an Olympus BX43 microscope (Olympus Corporation, Tokyo, Japan).

### 2.3 Isolation, purification of bacteria and microscopic examination by Gram staining

Milk cistern fluid samples from affected goats were thawed at room temperature and inoculated onto Nutrient Agar plates. After spread-plating, the plates were incubated at 37 °C for 24 h in a constant-temperature incubator. Predominant colonies were selected and subcultured onto Baird-Parker Agar, Columbia CNA Agar with 5% Sheep Blood, *Staphylococcus aureus* Chromogenic Medium, Nutrient Agar, and MacConkey Agar (Qingdao Hope Bio-Technology Co., Ltd., Qingdao, Shandong, China). These plates were then incubated at 37 °C for 24 h. Colony morphology, size, and color were observed to confirm the predominant colony types. Single colonies of the predominant type(s) were inoculated into Nutrient Broth for enrichment. Following enrichment, the broth cultures were repeatedly streaked onto the aforementioned selective and non-selective media. This purification process was iterated until the morphology of all the colonies on the plate is exactly the same. Three to six typical colonies were selected for Gram staining and microscopic examination.

### 2.4 Biochemical identification tests

Typical purified colonies were picked and inoculated into bacterial miniaturized biochemical test tubes containing melibiose, mannitol, N-acetylglucosamine, mannose, fructose, maltose, lactose, and trehalose, as well as into a coagulase test using rabbit plasma (Qingdao Hope Bio-Technology Co., Ltd., Qingdao, Shandong, China). All tests were incubated at 35 °C for 24 h in a constant-temperature incubator. Biochemical reaction results were recorded after the incubation period.

### 2.5 16S rDNA gene sequencing and identification

Genomic DNA was extracted from the bacterial isolates using a Bacterial DNA Extraction Kit (Solarbio Science & Technology Co., Ltd., Beijing, China). The 16S rDNA gene was amplified by polymerase chain reaction (PCR) using the 2 × GSTaq PCR Mix Kit (Beijing Jinshen Biotechnology Co., Ltd., Beijing, China). The PCR products were then electrophoresed on a 2% agarose gel for verification. DNA bands were visualized and photographed using a GenoSens1880 Gel Imaging System (Qinxiang Scientific Instrument Co., Ltd., Shanghai, China). The specific target DNA band was excised from the agarose gel under UV illumination. The amplified DNA fragment was purified from the gel slice using an Agarose Gel DNA Recovery Kit (Solarbio Science & Technology Co., Ltd., Beijing, China). The purified PCR products were preserved and submitted to Shanghai Biosune Biotechnology Co., Ltd. (Shanghai, China) and Shanghai Sangon Biotech Co., Ltd. (Shanghai, China) for bidirectional Sanger sequencing. Following assembly with Seqman software, the resulting sequences were subjected to BLAST analysis against the GenBank database on the NCBI website to determine their phylogenetic affiliations. Subsequently, MEGA 11.0 software was applied for multiple sequence alignment to construct a phylogenetic tree and identify the species of bacteria.

### 2.6 Animal challenge experiment

A total of 20 healthy Kunming mice (10 females weighing 34.0 ± 0.5 g and 10 males weighing 40.0 ± 0.5 g) were used. The mice were randomly divided into a control group and a challenge group (*n* = 10 each), with each group comprising five mice of each sex. Mice in the challenge group received an intraperitoneal injection of 0.5 mL of a bacterial suspension adjusted to 20 McFarland standard turbidity (approximately 6 × 10^9^ CFU/mL). The infection dose was determined through the pre-experiment of gradient concentration in the early stage. The results of the pre-experiment indicated that this dose could establish a stable bacterial load in the organs of mice within 24 h. Control mice were injected intraperitoneally with an equal volume of sterile physiological saline. After the infected group showed clinical symptoms, all mice were euthanized via cervical dislocation and subjected to necropsy. Euthanasia procedures were followed based on the Chinese Association for Laboratory Animal Sciences guidelines. All protocols were approved by the Institutional Animal Care and Use Committee of Yichun University (Approval No. JXSTUDKY2022009). Tissue samples from the heart, liver, spleen, lung, and kidney were aseptically collected for bacterial re-isolation culture. Additionally, representative lesions observed in these organs were fixed in 10% neutral buffered formalin, processed for histopathological examination by hematoxylin and eosin (H&E) staining, and examined microscopically as described in the previous section.

### 2.7 Preparation of susceptibility disks for antibiotics, CHMs, and phytogenic extracts

#### 2.7.1 Antibiotics

Susceptibility disks for 30 Antibiotics were commercially obtained (Hangzhou Microbial Reagent Co., Ltd., Zhejiang, China) and used as per the manufacturer’s instructions.

#### 2.7.2 CHMs

Eighteen CHMs (Kangmei Pharmaceutical Co., Ltd., Guangzhou, China) were individually processed. For each herb, 40 g was placed in a beaker, soaked in 400 mL of ultrapure water for 60 min, and then decocted by boiling vigorously followed by simmering for 60 min. The decoction was filtered through gauze. The residue was subjected to a second extraction with an additional 400 mL of ultrapure water, using the same boiling and simmering procedure for 60 min, followed by filtration. The two filtrates were combined and concentrated under reduced pressure to a final volume of 40 mL, achieving a concentration equivalent to 1 g crude herb per mL. The concentrated decoction was sterilized by autoclaving at 121 °C for 30 min. Sterile filter paper disks (6 mm in diameter) were immersed in the sterile decoction for 24 h. The impregnated disks were then dried and stored at 4 °C for later use.

#### 2.7.3 Phytogenic extracts

Fifteen phytogenic extracts (Shanghai Aladdin Biochemical Technology Co., Ltd., Shanghai, China) were individually prepared. For each extract, 2 mg was dissolved in 100 μL of dimethyl sulfoxide (DMSO) to yield a stock solution of 20 mg/mL. This solution was sterilized by filtration through a 0.22 μm pore-size membrane filter. Sterile filter paper disks (6 mm in diameter) were immersed in the sterile extract solution for 24 h. The impregnated disks were then dried and stored at 4 °C for later use.

### 2.8 Kirby-Bauer (K-B) disk diffusion susceptibility test

A bacterial suspension of the purified isolate was prepared in physiological saline and adjusted to a turbidity equivalent to the 0.5 McFarland standard. This suspension was uniformly swabbed onto the surface of a Mueller-Hinton (MH) agar plate (Qingdao Hope Bio-Technology Co., Ltd., Qingdao, Shandong, China). Susceptibility disks were aseptically placed onto the inoculated agar surface. The plate was then incubated at 37 °C under aerobic conditions for 16–18 h. Following incubation, the diameters of the zones of inhibition (D) around each disk were measured using digital calipers and recorded.

## 3 Results

### 3.1 Histopathological findings from H&E staining

**Figure 1:**
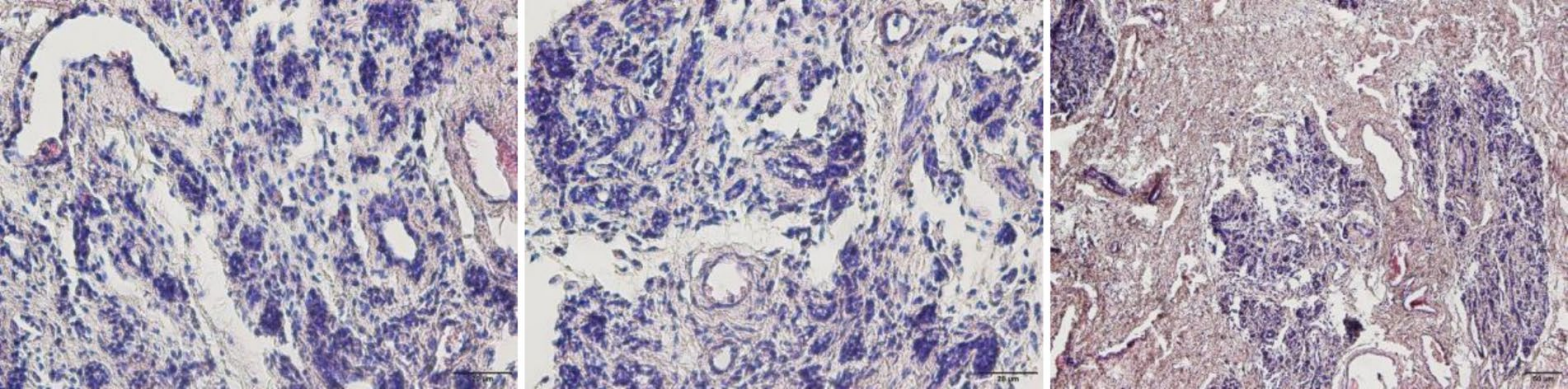
Hematoxylin and eosin (H&E) staining of pathological breast tissue (400×).

Histopathological examination of H&E-stained sections revealed fibrosis of the mammary gland tissue, atrophy of mammary acini, extensive inflammatory cell infiltration, and multifocal granulomas in the dairy goats with mastitis (Figure 1).

### 3.2 Colonial morphology and microscopic examination

**Figure 2:**
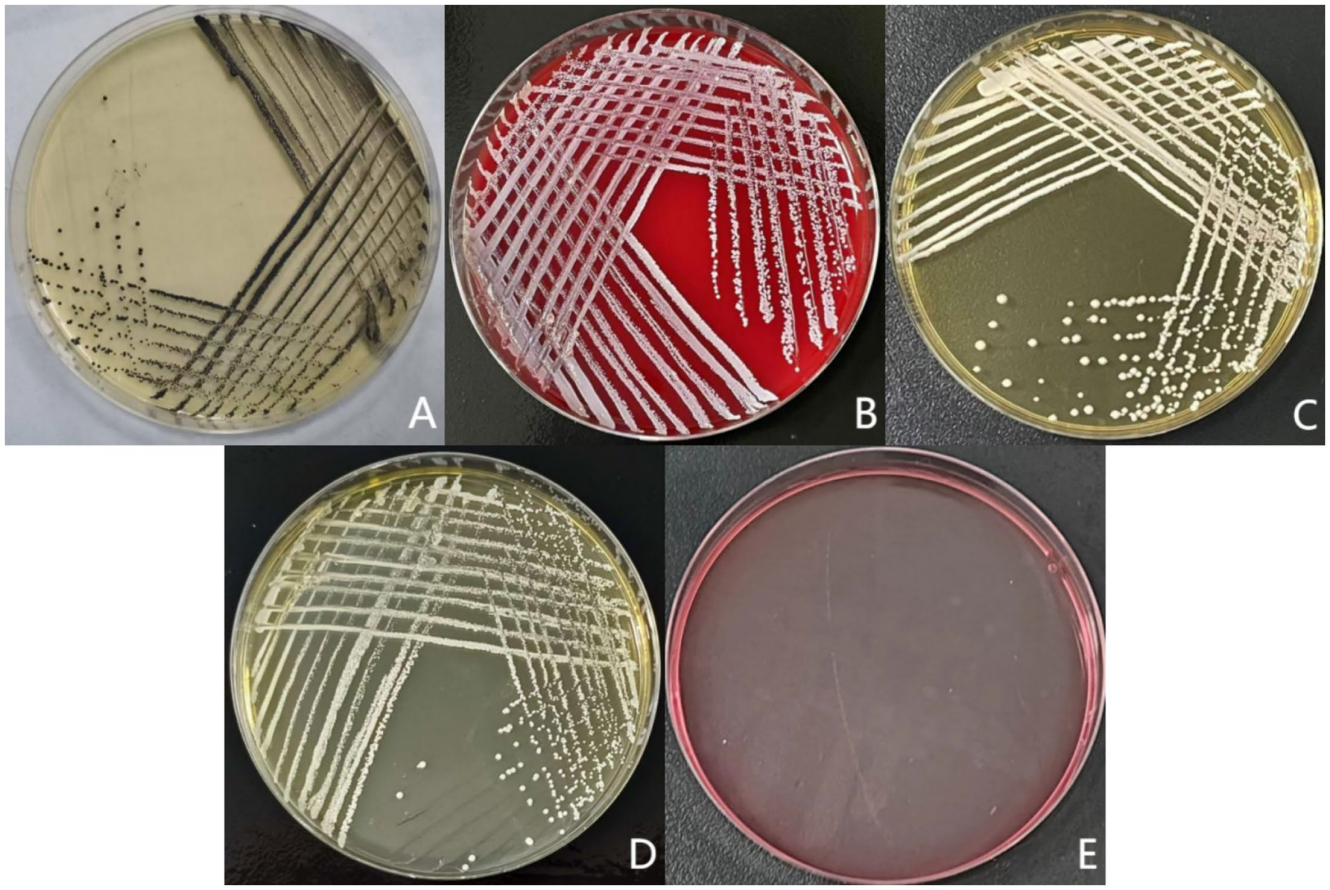
Colony morphology on differential media results. **(A)** Baird-Parker agar medium; **(B)** Columbia CNA blood agar plate; **(C)** Chromogenic medium for *Staphylococcus aureus*; **(D)** General nutrient agar medium; **(E)** MacConkey agar medium.

As shown in Figure 2, the purified isolate exhibited distinct colonial morphologies on various media: On Baird-Parker agar, black, circular colonies with smooth, convex surfaces and no surrounding halo were observed. On Columbia CNA agar with 5% sheep blood, small, white, circular colonies displaying gamma-hemolysis (γ-hemolysis) were present. On *Staphylococcus aureus* chromogenic medium, opaque, white, circular colonies grew. On nutrient agar, white, circular colonies with smooth, convex surfaces were formed. No growth was observed on MacConkey agar.

**Figure 3:**
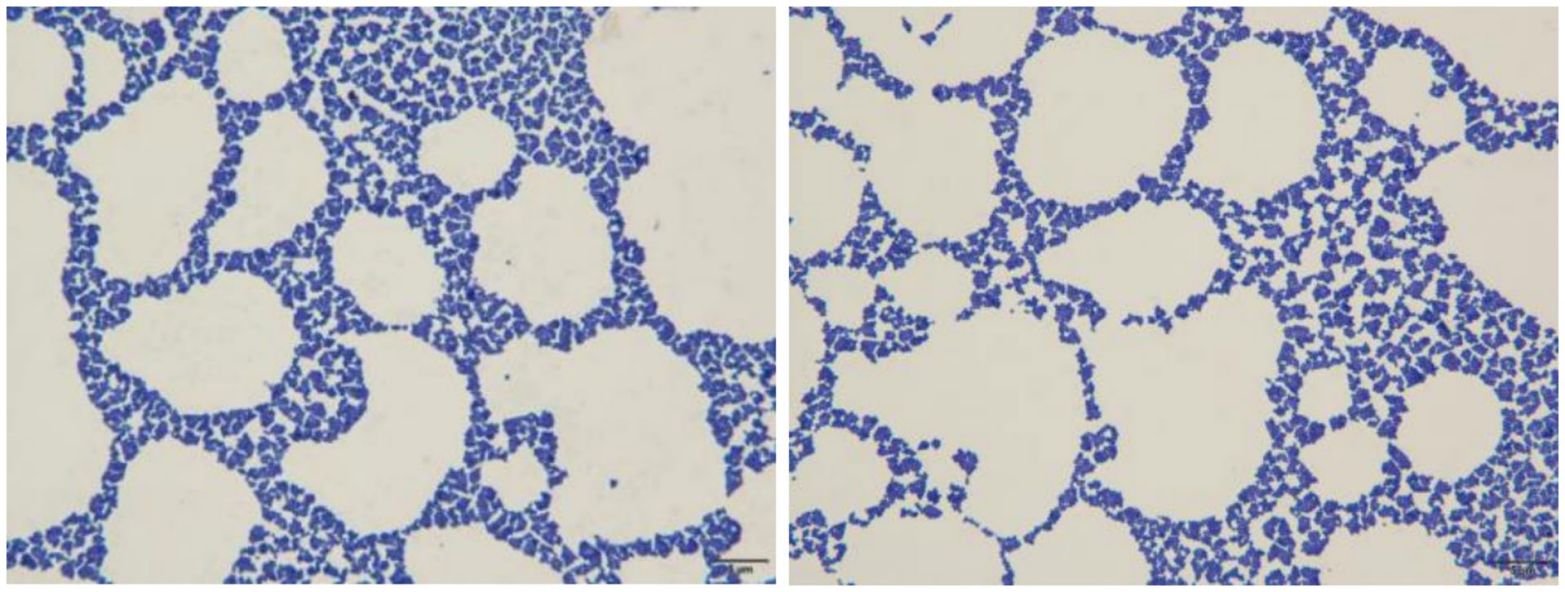
Gram staining results. Isolated bacteria from Columbia CNA blood agar plate (1,000×).

As shown in Figure 3, Gram staining of representative purified colonies revealed Gram-positive cocci arranged in characteristic grape-like clusters. The cells lacked visible spores, flagella, and a distinct capsule. Based on these morphological and staining characteristics, the isolate was identified as a Gram-positive bacterium belonging to the genus *Staphylococcus*.

### 3.3 Biochemical characterization

Following 24-h incubation at 35 °C in biochemical test tubes, the isolate exhibited: Positive reactions for: Mannose, Fructose, Maltose, Lactose, Trehalose, Sucrose, Urea. Negative reactions for: Melibiose, Mannitol, N-Acetylglucosamine, Xylose, Arginine Hydrolysis, Xylitol, Sorbitol. Coagulation of rabbit plasma was negative (Table 1). These biochemical profiles identified the isolate as coagulase-negative staphylococci.

**Table 1:** Biochemical characterization of bacterial isolates.

Items	1	2	3
Melibiose	−	−	−
Mannitol	−	−	−
Mannose	+	+	+
N-acetylglucosamine	−	−	−
Fructose	+	+	+
Xylose	−	−	−
Arginine hydrolysis	−	−	−
Xylitol	−	−	−
Malt sugar	+	+	+
Lactose	+	+	+
Trehalose	+	+	+
Sucrose	+	+	+
Urea	+	+	+
Sorbitol	−	−	−

“+” positive; “−” Negative.

### 3.4 16S rDNA gene sequencing identification

**Figure 4:**
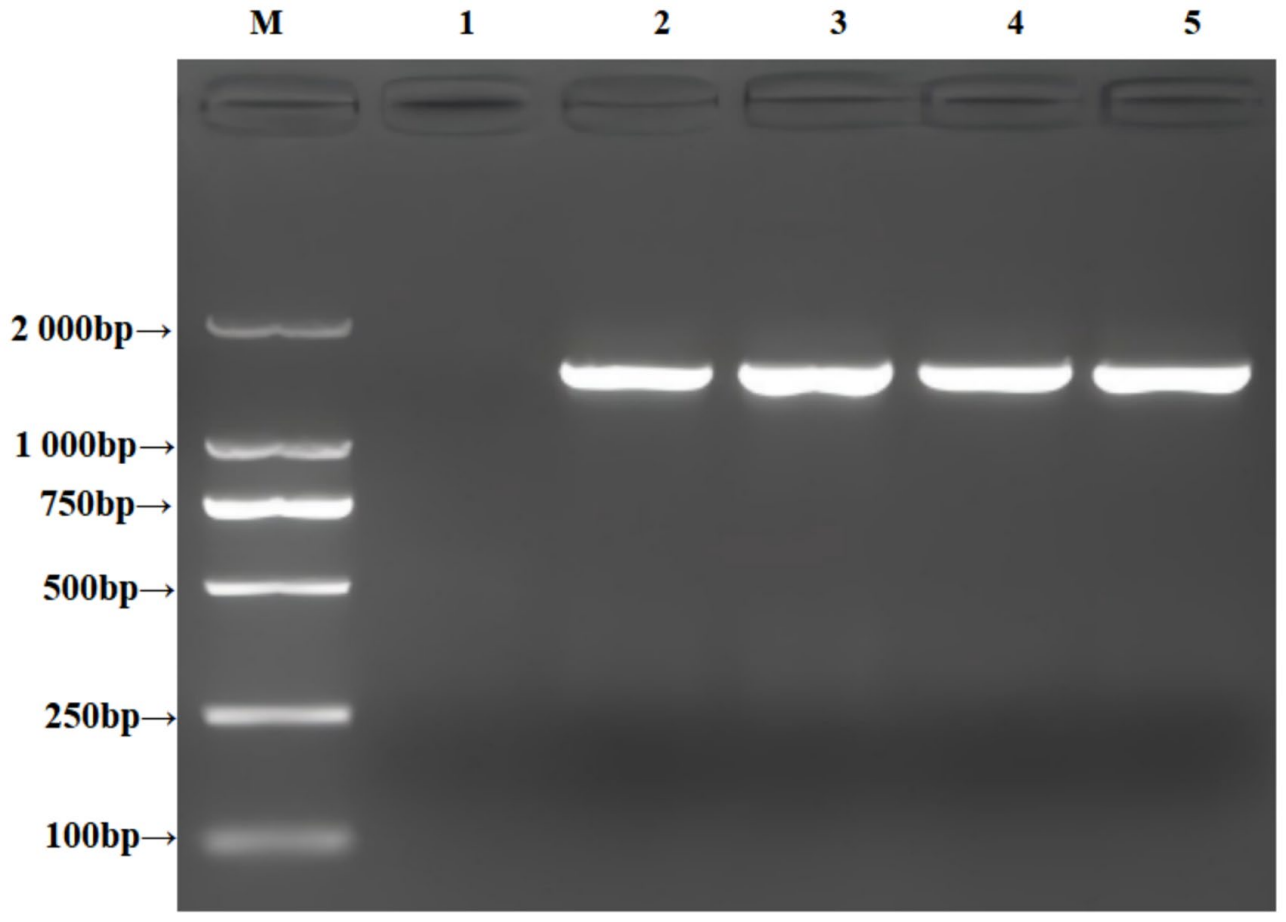
Electrophoresis of PCR products from isolated strains (M: DL2000 DNA marker; 1: Blank control; 2–5: Bacterial isolates).

**Figure 5:**
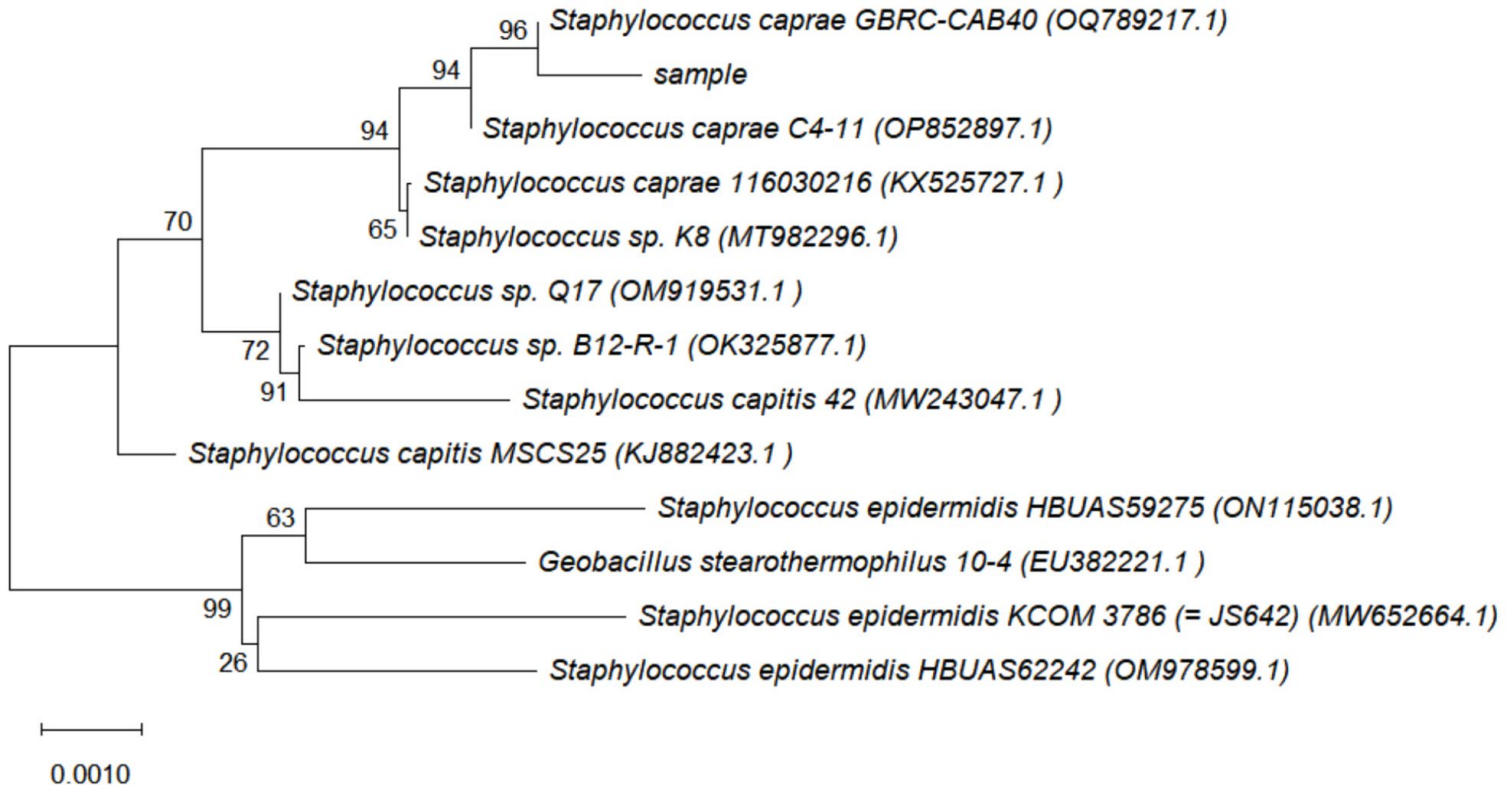
16S rDNA gene phylogenetic tree. The number in parentheses is the GenBank accession number; The numbers at nodes represent the confidence values of bootstrap test with 1,000 repeated samples. 0.001 in the distance scale indicates units of genetic distance.

DNA extracted from representative colonies was amplified by PCR and subjected to agarose gel electrophoresis. Gel documentation revealed a distinct band at 1,500 bp (Figure 4). The obtained sequences from the sequencing of the recovered products were assembled using Seqman, followed by homology comparison via NCBI-BLAST. Multiple sequence alignment was performed using MEGA 11.0 software to construct a phylogenetic tree (Figure 5). The results demonstrated that the isolated strain clusters within the same evolutionary branch as *S. caprae* strain GBRC-CAB40, with high sequence similarity, further confirming that the strain isolated in this study is *S. caprae*.

### 3.5 Virulence of *Staphylococcus caprae* in mice

**Figure 6:**
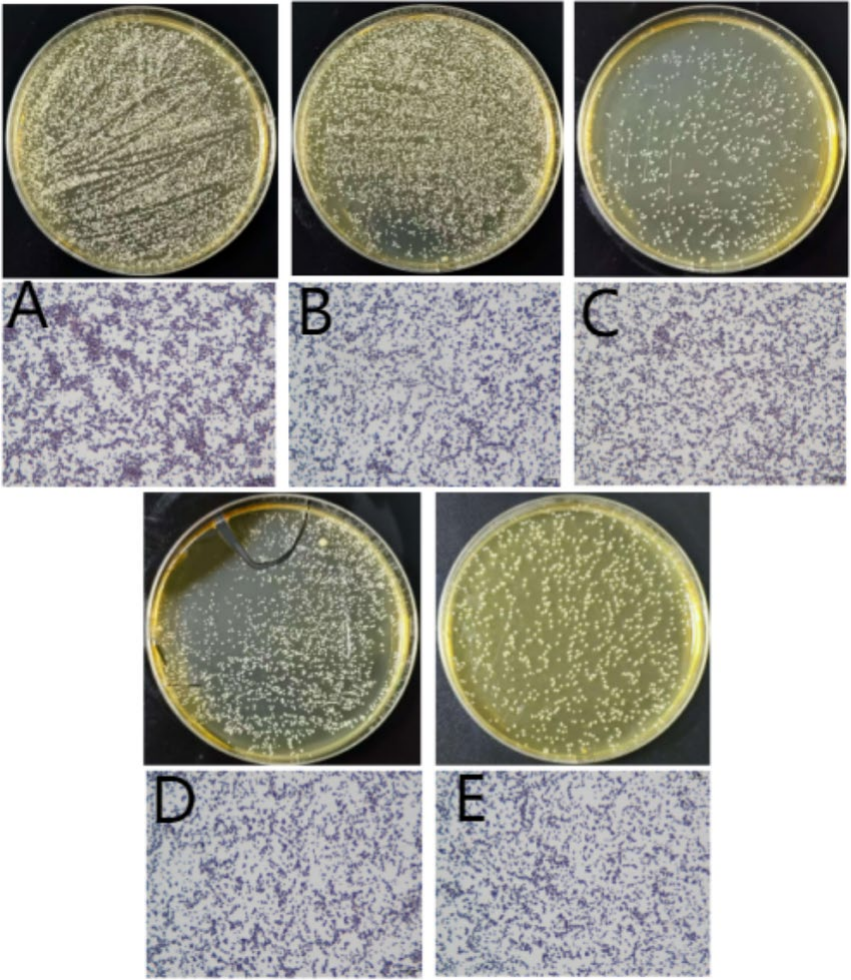
Bacteria isolated from various mouse organs and their corresponding Gram staining microscopy images (400×). **(A)** Liver, **(B)** spleen, **(C)** lung, **(D)** kidney, **(E)** heart.

**Figure 7:**
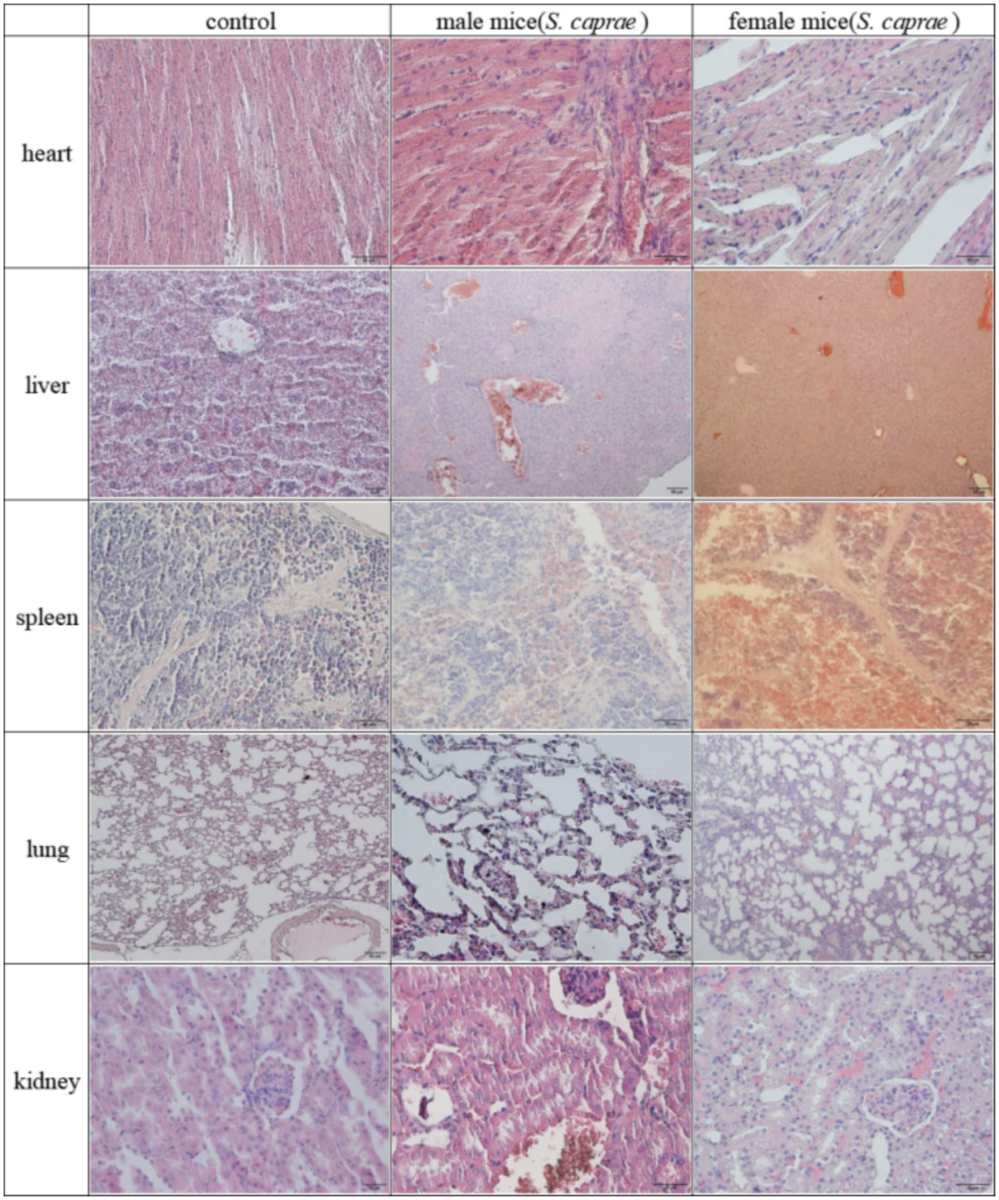
Histopathological examination of mouse organs by H&E staining (400×).

The mice in the infection group showed mental depression, while controls remained normal. Bacterial isolates were recovered from all major organs (heart, liver, spleen, lung, kidney) (Figure 6). Histopathological analysis revealed multi-organ damage: cardiac tissue showed inflammatory infiltration; hepatic sections demonstrated hepatocellular swelling and necrotic congestion; pulmonary lesions featured alveolar epithelial hyperplasia with interstitial thickening and focal collapse; splenic pathology included marginal hemorrhage and sinus obliteration; renal damage manifested as tubulointerstitial fibrosis, epithelial desquamation, and peritubular inflammation (Figure 7).

### 3.6 Antimicrobial susceptibility profiling

#### 3.6.1 Antibiotics susceptibility of *Staphylococcus caprae*

All tested antibiotics inhibited *S. caprae* growth with varying efficacy. High susceptibility was observed to: β-lactams: Cefazolin, Cefradine, Cephalexin, Cefuroxime, Fluoroquinolone: Ciprofloxacin. Conversely, poor susceptibility occurred with Penicillin, Ampicillin, Vancomycin, and Polymyxin B (Table 2).

**Table 2:** Results of drug susceptibility test of antibiotics against *S. caprae.*

Drugs name (*n* = 3)	Antimicrobial zone diameter Mean (mm) ± SD
Cefazolin	43.3 ± 0.46
Cefradine	48.3 ± 0.4
Cephalexin	46.3 ± 0.63
Cefuroxime	44.3 ± 0.45
Ceftriaxone sodium	33.7 ± 0.49
Ceftazidime	24.7 ± 0.3
Cefoperazone	28.7 ± 0.58
Piperacillin	26.7 ± 0.58
Penicillin	19.3 ± 0.64
Oxacillin	29.7 ± 0.35
Ampicillin	23.3 ± 0.8
Carbenicillin	31.3 ± 0.8
Kanamycin	35.3 ± 0.5
Ofloxacin	37.0 ± 0.36
Neomycin	33.3 ± 0.5
Amikacin	32.7 ± 0.42
Tetracyclines	34.0 ± 0.25
Doxycycline	38.0 ± 0.35
Minocycline	35.7 ± 0.4
Erythromycin	33.3 ± 0.46
Aboren	29.0 ± 0.26
Clindamycin	32.0 ± 0.53
Vancomycin	22.7 ± 0.4
Pediatric compound sulfamethoxazole tablets	36.0 ± 0.4
Polymyxin B	22.0 ± 0.35
Chloroamphenicol	33.7 ± 0.35
Furazolidone	27.7 ± 0.32
Ciprofloxacin	41.3 ± 0.5
Gentamicin	36.7 ± 0.58
Norfloxacin	36.7 ± 0.45

“D” Antimicrobial zone diameter. D < 10 mm, Resistant; 10 mm ≤ D ≤ 15 mm, Intermediate; D > 15 mm, Susceptible.

#### 3.6.2 CHMs susceptibility of *Staphylococcus caprae*

*S. caprae* exhibited: Susceptibility: *Rhus chinensis* (Galla Chinensis), *Coptis chinensis* (Chinese Goldthread). Intermediate susceptibility: *Punica granatum* (Granati Pericarpium), *Scutellaria baicalensis* (Scutellariae Radix), *Terminalia chebula* (Medicine Terminalia Fruit), *Forsythia suspensa* (Forsythia Fruit). Resistance: 12 additional CHMs including *Glycyrrhiza uralensis* (Glycyrrhizae Radix), *Prunus mume* (Smoked Plum), *Trichosanthes kirilowii* (Snakegourd Fruit) (Table 3).

**Table 3:** Results of drug susceptibility test of CHMs against *S. caprae.*

Drugs name (*n* = 3)	Antimicrobial zone diameter Mean (mm) ± SD
*Trichosanthes kirilowii*	6.0 ± 0
*Glycyrrhiza uralensis*	8.0 ± 0
*Pulsatilla chinensis*	6.0 ± 0
*Viola philippica*	6.0 ± 0
*Carthamus tinctorius*	6.0 ± 0
*Prunus mume*	6.3 ± 0.06
*Leonurus japonicus*	6.0 ± 0
*Punica granatum*	15.0 ± 0.1
*Rhus chinensis*	24.0 ± 0
*Houttuynia cordata*	6.0 ± 0
*Lonicera japonica*	6.0 ± 0
*Taraxacum mongolicum*	6.0 ± 0
*Terminalia chebula*	12.2 ± 0.03
*Chrysanthemum morifolium*	6.0 ± 0
*Scutellaria baicalensis*	14.7 ± 0.15
*Coptis chinensis*	22.0 ± 0
*Paeonia lactiflora*	6.0 ± 0
*Forsythia suspensa*	10.7 ± 0.12

“D” Antimicrobial zone diameter. D < 10 mm, Resistant; 10 mm ≤ D ≤ 15 mm, Intermediate; D > 15 mm, Susceptible.

#### 3.6.3 Phytogenic extracts susceptibility of *Staphylococcus caprae*

*S. caprae* showed different susceptibilities to all the tested phytogenic extracts: Susceptibility: Berberine, Gallic acid, Tannic acid, Sodium houttuyfonate, Punicalagin. Intermediate susceptibility: Shikonin, Resveratrol, Naringenin, Baicalein, Chlorogenic acid, Caffeic acid, Quercetin, Phillyrin. Resistance: Pulsatilla saponin, Curcumin (Table 4).

**Table 4:** Results of drug susceptibility test of phytogenic extracts against *S. caprae.*

Drugs name (*n* = 3)	Antimicrobial zone diameter Mean (mm) ± SD
Sodium houttuyfonate	16.0 ± 0.17
Berberine	20.0 ± 0.2
Baicalein	13.2 ± 0.08
Resveratrol	14.5 ± 0.06
Chlorogenic acid	12.0 ± 0
Quercetin	10.7 ± 0.06
Gallic acid	20.0 ± 0.17
Naringenin	14.3 ± 0.06
Phillyrin	10.2 ± 0.08
Shikonin	15.0 ± 0.1
Tannic acid	16.3 ± 0.15
Pulsatilla saponin	8.7 ± 0.06
Caffeic acid	12.0 ± 0
Curcumin	8.2 ± 0.12
Punicalagin	15.5 ± 0.09

“D” Antimicrobial zone diameter. D < 10 mm, Resistant; 10 mm ≤ D ≤ 15 mm, Intermediate; D > 15 mm, Susceptible.

## 4 Discussion

A primary challenge confronting the modern dairy goat industry is the minimization of mastitis incidence. The identification of mastitis pathogens provides crucial bacteriological information and facilitated the development of more effective disease control strategies. This study determined the predominant bacterial strain isolated from clinical samples, thereby expanding the epidemiological data on mastitis-causing pathogens in dairy goats from the Western of Jiangxi Province, China. Furthermore, the susceptibility of *S. caprae* to antibiotics, Chinese herbal medicines, and phytogenic extracts was evaluated. Through *in vitro* antibacterial assays, the abundant local resources of Chinese herbal medicines and phytogenic extracts can be transformed into economical, effective, and sustainable tools for mastitis control, thereby reducing dependence on antibiotics. This will ultimately help farmers in developing regions bolster their income, safeguard food safety, and promote the sustainable development of the livestock industry.

Histopathological examination using hematoxylin and eosin (H&E) staining revealed characteristic tissue lesions, including extensive inflammatory cell infiltration, mammary tissue fibrosis, and multiple granulomas. These findings align with the clinicopathological outcomes of mastitis observed in bacterial challenge experiments reported by Singh et al. (16). Chronic non-suppurative mastitis has been shown to be significantly associated with Coagulase-negative staphylococci (CNS) infections (17). In this case, affected dairy goats exhibited mammary tissue atrophy, fibrosis, hardening, and nodular formations. The condition was only detected by the owner at advanced stages, suggesting the likelihood of chronic persistent mastitis. Given that *S. caprae* is a CNS species, these observations further indicate its potential threat in subclinical mastitis presentations.

In bacteriological identification, Abdalhamed et al. isolated bacteria from milk samples by streaking on sheep blood agar, followed by subculturing on selective media including mannitol salt agar, Salmonella-Shigella agar, Edwards medium, and MacConkey agar. Pathogens were ultimately diagnosed based on colony morphology, pigmentation, and hemolytic characteristics (18). This approach is similar to the colony morphology identification using five media employed in the present study, collectively providing enhanced reference standards for subsequent morphological assessments. Praja et al. identified *Staphylococcus aureus* based on growth and fermentation attributes on mannitol salt agar, Gram staining, and biochemical tests confirming catalase positivity, oxidase negativity, Voges-Proskauer positivity, coagulase positivity, and urease positivity (19). Similarly, Okoko et al. identified staphylococci based on growth and fermentation characteristics on mannitol salt agar and catalase positivity, subsequently differentiating *S. aureus* from CNS using rabbit plasma and coagulase tests (20). Building upon these methods, this study incorporated additional biochemical identification assessing bacterial utilization of substrates such as sugars, amino acids, and enzymes. Biochemical reactions were determined based on observable changes. Combined with a negative rabbit plasma test result, the isolate was confirmed as CNS. However, numerous possibilities for the specific predominant bacterial species remained (21), rendering the identification insufficient for guiding targeted farm management improvements. 16S rRNA detection is recognized as a more reliable and reproducible method for identifying CNS species causing caprine mastitis (22). In this study, 16S rDNA analysis identified *S. caprae* as the predominant bacterium isolated from mastitic mammary tissue samples of dairy goats. Notably, CNS species accounted for nearly 80% of pathogens detected in goat mastitis cases in Slovakia (23) and Italy (24), with *S. caprae* consistently observed as the most prevalent specific pathogen, followed by *Staphylococcus epidermidis*. Both species are frequently associated with chronic persistent mastitis, coinciding with the pathogen identification results and clinical manifestations observed in this study.

Our findings reveal a distinct *in vitro* antimicrobial susceptibility profile for the *S. caprae* isolate. Consistent with prior reports by Moroni et al. (25), β-lactam antimicrobials demonstrated high efficacy overall. Specifically, cephalosporins-including Cefazolin, Cefradine, Cephalexin, Cefuroxime-were the most sensitive agents in this study. This aligns with Moroni et al.’s observation that most β-lactams, except Cefoperazone, were effective against *S. caprae* (25). Fluoroquinolone susceptibility (14) is consistent with the Ciprofloxacin susceptibility observed here. Notably, while Moroni et al. identified benzylpenicillin (penicillin G) as the most potent single agent against *S. caprae*, with amoxicillin (alone or combined with clavulanic acid) also exhibiting high activity (24), a significant reduction in susceptibility to these agents (penicillin, ampicillin) was observed in our isolate. This discrepancy may be attributed to the potential development and enrichment of resistance genes, likely associated with prolonged on-farm usage of penicillin-class drugs by the owner. This hypothesis is supported by studies documenting higher ampicillin resistance frequencies in CNS isolated from goats with subclinical mastitis, suggesting a link between exposure history and resistance development within related species occupying the same ecological niche (26, 27). Furthermore, the observed poor susceptibility to vancomycin, polymyxin B, and furazolidone underscores the critical importance of susceptibility testing to guide therapy. Crucially, our results confirm the poor efficacy of tetracycline (24). Collectively, these findings highlight the imperative for prudent antimicrobial stewardship within livestock settings to preserve the efficacy of antibiotics like β-lactams. Or it can be used in combination with CHMs to reduce the pressure of bacterial resistance (28).

Studies indicate that CHMs and their phytogenic extracts can ameliorate the pathological progression of subclinical mastitis through multiple pathways. First, they exert anti-inflammatory, antioxidant, and immunomodulatory properties via metabolomic regulation (29). Second, they suppress mammary inflammatory responses and pyroptosis by modulating the NF-κB pathway and NLRP3 inflammasome (30). Third, they exhibit significant antimicrobial activity against multidrug-resistant strains and effectively disrupt biofilm structures (31). Finally, they mitigate mastitis occurrence by modulating the gut microbiota (32). These mechanisms substantiate CHMs and phytogenic extracts as viable alternative strategies to conventional antimicrobials. *In vitro* susceptibility testing in this study revealed that among 18 tested CHMs, *Rhus chinensis* (Galla Chinensis), *Coptis chinensis* (Chinese Goldthread), *Punica granatum* (Granati Pericarpium), and *Scutellaria baicalensis* (Scutellariae Radix) demonstrated the most potent inhibitory effects. Among 15 phytogenic extracts, Berberine, Gallic acid, Tannic acid, Sodium houttuyfonate, and Punicalagin exhibited prominent activity. Notably, a clear correspondence exists between the antibacterial effects of the source herbs and their core bioactive constituents: Gallic acid and Tannic acid for *Rhus chinensis*, Berberine (the primary alkaloid) for *Coptis chinensis*, and Punicalagin (a polyphenol) for *Punica granatum*. This efficacy concordance between bioactive constituents and parent herbs validates the antibacterial mechanisms of CHMs at the molecular level, providing a scientific basis for their clinical application. Furthermore, the *ica* operon is recognized as crucial for regulating biofilm formation in *S. caprae* (33, 34). Convergent evidence shows that *Rhus chinensis* significantly inhibits *ica* gene expression (35), Sodium houttuyfonate can synergize with subinhibitory concentrations of erythromycin to inhibit the formation of biofilms and the expression of icaA (36), and the CHM formulation San-Huang Decoction [*Coptis chinensis*, *Scutellaria baicalensis*, *Cortex Phellodendri Chinensis* (Phellodendron chinense)] downregulates *icaAD* gene expression (37), both suppressing biofilm formation in antibiotic-resistant *Staphylococci*-aligning with the susceptibility results of this study. This suggests the *ica* operon may represent a key therapeutic target for CHMs and phytogenic extracts. Furthermore, Berberine, Gallic acid, Tannic acid and Punicalagin may inhibit mastitis pathogens by disrupting the lipid bilayer and microbial cell membranes, damaging cell walls, and suppressing bacterial growth and extracellular microbial enzymes (38–41). However, efficacy *in vitro* does not necessarily translate to efficacy in animal models. Rigorous *in vivo* clinical trials are essential to determine appropriate dosage, route of administration, and to evaluate safety and residue levels in milk (42). Future work will investigate the therapeutic effects of antibiotics, CHMs, and phytogenic extracts—both individually and in combination—using animal infection models.

Although previous studies have raised concerns about the potential zoonotic transmission of *S. caprae* (14, 15), our current focus on its *in vitro* antibacterial activity did not directly investigate this aspect. Nevertheless, observations from this study suggest that the actual zoonotic transmission risk appears to be minimal.

## 5 Conclusion

This study confirms that the dairy goat farm in western Jiangxi Province is experiencing persistent challenges with *Staphylococcus caprae* (*S. caprae*) infection. Furthermore, the pathogen exhibits a dual evolutionary trajectory toward enhanced antimicrobial resistance and increased pathogenicity. Therefore, the targeted and rational use of antibiotics is imperative to mitigate t he development of resistance in *S. caprae*. Concurrently, consideration should be given to alternative or complementary therapies utilizing Chinese herbal medicines (CHMs) such as *Rhus chinensis* (Galla Chinensis), *Coptis chinensis* (Chinese Goldthread), *Punica granatum* (Granati Pericarpium), and *Scutellaria baicalensis* (Scutellariae Radix), or their phytogenic extracts including Berberine, Gallic acid, Tannic acid, Sodium houttuyfonate, and Punicalagin. Future research will focus on the molecular basis of drug resistance mechanisms and virulence evolution in *S. caprae*, systematically evaluate the *in vitro* and *in vivo* antibacterial effects of CHMs, and explore their synergistic interactions with conventional antibiotics, so as to facilitate the development of more applicable integrated strategies for disease control.

## Data Availability

The data presented in the study are deposited in the NCBI repository, accession number PX599637.1.
